# Episodic biliary obstruction due to an intrahepatic biliary cystadenoma: a case report

**DOI:** 10.4076/1752-1947-3-9032

**Published:** 2009-09-08

**Authors:** Pulathis N Siriwardana, Aloka Pathirana

**Affiliations:** 1 University Surgical Unit, Colombo South Teaching Hospital, Kalubowila, Sri Lanka; 2 Department of Surgery, Faculty of Medical Sciences, University of Sri Jayawardanapura, Sri Lanka

## Abstract

**Introduction:**

Biliary cystadenoma is a rare, benign neoplasm of the bile ducts with malignant potential. Symptoms, predominantly right hypochondrial pain and the feeling of a lump or fullness are usually due to the mass effect. Jaundice is rare. This is the fifth reported patient with an intrahepatic biliary cystadenoma giving rise to episodic biliary obstruction, which is usually caused by choledocholithiasis or periampullary carcinoma. Considering the mean age of previous similar patients (53.5, standard deviation 14.6 years), the early age of presentation is very unusual in our patient.

**Case presentation:**

A 25-year-old Asian woman presented with right hypochondrial pain and episodic biliary obstruction. Contrast enhanced computed tomography revealed a cystic mass in segment 4B and protruding into and along the left hepatic duct. Laparotomy confirmed the contrast enhanced computed tomography findings and histology revealed an intrahepatic mucinous biliary cystadenoma.

**Conclusion:**

Biliary cystadenoma should be considered as a differential diagnosis in patients with cystic liver lesions who present with episodic biliary obstruction. Due to the reported malignant potential, radical surgery such as wide local excision of the lesion or hepatic resection is needed to minimize the risk of local recurrence.

## Introduction

Biliary cystadenoma (BCA) is a rare, benign cystic lesion of the liver, arising from the biliary ducts, typically lined with columnar epithelium, and usually with an "ovarian like" cellular stroma. The cyst is usually multilocular. A majority arise from the intrahepatic ducts, predominantly within the left lobe [1]. They occur mostly in middle aged women. The diameter can vary from 2 to 30 cm (mean 15 cm) and symptoms such as right hypochondrial pain and abdominal lump are mass effects by virtue of size [1,2].

## Case presentation

A 25-year-old Sri Lankan woman was referred to our unit with a preliminary ultrasound scan (USS) suggestive of a liver cyst. She had right hypochondrial pain, and several episodes of intractable itching. Only one of these episodes of itching was associated with clinically and biochemically proven obstructive jaundice.

On examination, she was anicteric and did not have any features of liver disease. Abdominal examination was unremarkable.

**Figure 1:**
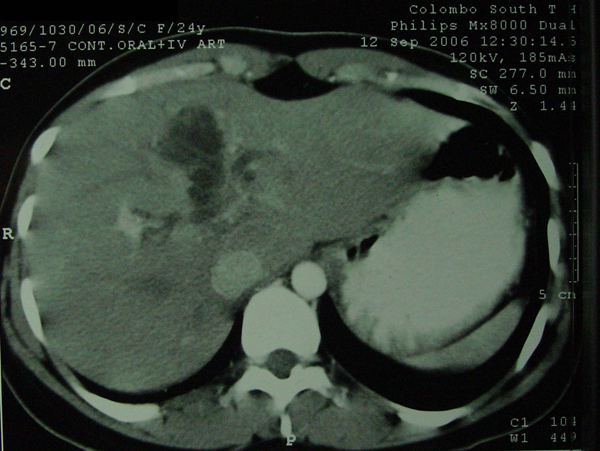
**Pre-operative contrast enhanced computed tomography of the abdomen performed 2 months before surgery, demonstrating the septate intrahepatic biliary cystadenoma with extension to the left hepatic duct**.

Liver function tests were normal. Serum tumor markers including carcinoembryonic antigen (CEA), α feto protein and CA 19-9 levels were within normal limits. An USS of the liver showed a 5.5 cm × 4 cm multiloculated intrahepatic cyst with several smaller cysts within. The intrahepatic ducts were slightly dilated, with the cyst probably extending into the proximal bile duct. Contrast enhanced computed tomography (CECT) confirmed the presence of the cyst in segment 4B of the liver which had an enhancing thin wall with multiple septa and no intracystic solid component (Figure 1). CECT also revealed intrahepatic, common hepatic and proximal common bile duct dilatation probably due to an extension of the lesion along the left hepatic duct. A hydroxy iminodiacetic acid (HIDA) scan excluded a direct communication of the cyst with the biliary tree. Serum echinococcus IgG levels were normal.

**Figure 2:**
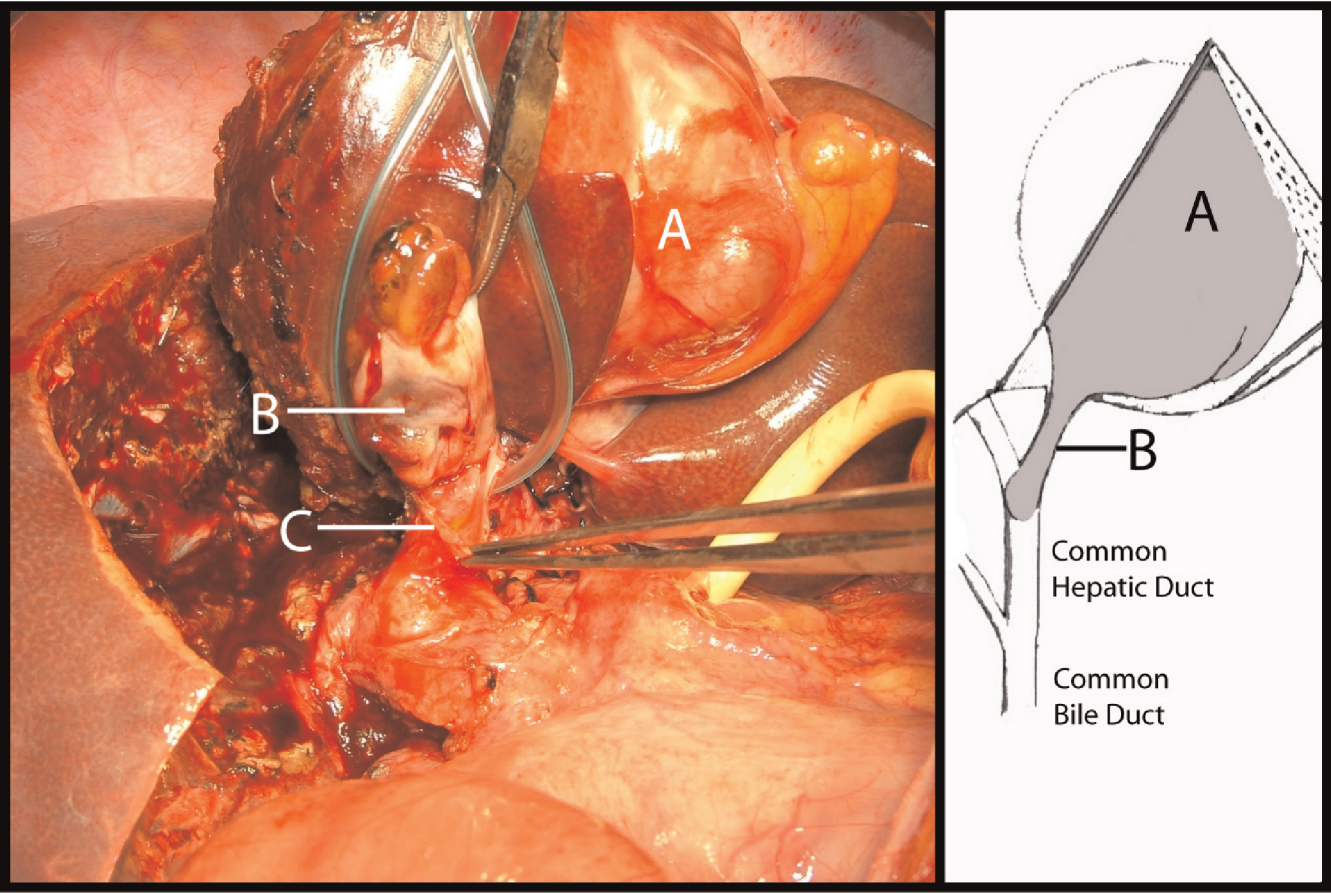
**Intra-operative image showing the partially resected intrahepatic biliary cystadenoma**. Partially resected left lobe with the intrahepatic biliary cystadenoma and its extension into the left hepatic duct which is cut open and the schematic diagram of the anatomical relationship of the intrahepatic biliary cystadenoma and the biliary ducts. The extrahepatic gall bladder, which is not shown in the image, was removed synchronously. **(A)** Intrahepatic biliary cystadenoma; **(B)** Polypoid extension of the intrahepatic biliary cystadenoma in the left hepatic duct (retracted upwards); **(C)** Opening of the left hepatic duct through which the extension of the intrahepatic biliary cystadenoma was extracted.

Laparotomy confirmed a lesion in the left side of the liver with an extension into the common hepatic duct along the left hepatic duct. The gallbladder was extrahepatic. During left hepatectomy, the cyst extension was removed intact, through the cut end of the left hepatic duct (Figure 2). A synchronous cholecystectomy was performed once the resection surface of the liver had been checked for biliary leaks by injecting saline through the cystic duct. The patient had an uneventful recovery.

Histology revealed a multilocular, mucinous biliary cystadenoma lined by a single layer of glandular epithelium, arising from the left intrahepatic ducts. A basement membrane separated the epithelial lining from the underlying ovarian type mesenchyme. The patient is well at 24 months with no evidence of recurrent disease.

## Discussion

There have been less than 100 cases of BCAs reported worldwide. These account for less than 5% of neoplasms originating from the bile duct [3].

BCAs are of two types, mucinous or serous. The more common mucinous type is subdivided by the presence or absence of a mesenchymal stroma between the inner epithelial lining and the outer basement membrane. Although both types can undergo malignant transformation to biliary cystadenocarcinoma (BCAC), absence of a mesenchymal stroma is known to be more aggressive, especially in men [1].

Jaundice is a rare presentation of BCA, and, to the best of our knowledge, episodic jaundice has only been reported in four patients [4,5]. There are only eight reported cases of intrahepatic BCA causing obstructive jaundice due to its extension into a major duct [4]-[7]. The most likely cause of episodic jaundice in this patient would have been recurrent hemorrhage into the cyst which was extending along the left hepatic duct to the confluence, causing a transient rise in intracystic pressure and in turn occlusion of the common channel of the extrahepatic biliary tree, as reported by Taketomi *et al.*[7].

Pre-operative diagnosis may be difficult. Generally on USS and CECT, they appear as focal lesions with internal septa, hence they may be confused with other cystic hepatic lesions such as a complicated cyst, mesenchymal hamartoma, undifferentiated embryonal sarcoma and cystic metastases in addition to the malignant counterpart of BCA and hydatid cyst [8]. While the BCAC has papillary excrescences with solid components, hydatid cysts are characterized by round and oval daughter cysts and "ring like" enhancement with contrast on CECT. Magnetic resonance imaging (MRI) also helps in diagnosis. In the presence of intralesional hemorrhage and hyperproteinous/mucinous contents, the distinction of BCA from BCAC may be difficult [6]. In the presence of obstructive jaundice, cholangiography, either endoscopic, percutaneous or magnetic resonance would show the level of obstruction. Since this patient's CECT revealed the cause of the biliary obstruction, cholangiography was not performed.

Treatment methods for BCA in the past have varied from aspiration, marsupialization, internal drainage, partial excision and enucleation [6,9]. The main concerns of these methods are local recurrence, malignant transformation and misdiagnosis of cancer. In addition, biliary peritonitis may occur following aspiration and marsupialization [9,10]. In the past, more conservative methods were favored probably due to poor pre-operative imaging and the lack of understanding of BCA's malignant potential. Enucleation may be possible provided there is no co-existing malignancy which can be detected by intra-operative ultrasound scan and frozen sections [9]. However, contemporary hepatic surgery has minimal morbidity and mortality [11]. Hence, as suggested by previous authors [6], we endorse formal liver resection or wide local excision as the treatment of choice for BCA.

## Conclusion

This article contributes to the medical literature by adding another reported case of episodic biliary obstruction due to an intrahepatic BCA protruding into the common hepatic duct, which may be considered as a very rare cause of intermittent jaundice. Pre-operative diagnosis may be confusing. Hence, a high degree of suspicion and a multidisciplinary approach are needed to plan the surgical procedure and prevent inadequate treatment. The surgical strategy would be to radically excise the lesion. A follow-up protocol is unavailable due to the small number of BCAs. However, we suggest long-term evaluation with clinical examination but more importantly with periodic ultrasound scans at least 3 monthly during the first year, 6 monthly for 2 years and annually subsequently to exclude local recurrence.

## Consent

Written informed consent was obtained from the patient for publication of this case report and any accompanying images. A copy of the written consent is available for review by the Editor-in-Chief of this journal.

## Competing interests

The authors declare that they have no competing interests.

## Authors' contributions

PNS is the principal and corresponding author. PNS actively managed the patient and was the first assistant to the surgeon (AP). PNS contributed to the paper by performing the literature survey, and interpreting and analyzing past cases to decide on management of the patient. PNS wrote the manuscript and edited the successive versions. AP is the senior author and the team surgeon. He was a major contributor in interpreting the cases in the literature and applying them to the management of the patient. AP contributed to the paper by planning the structure and editing successive versions. Both authors read and approved the final manuscript.
